# Self-Quarantine Noncompliance During the COVID-19 Pandemic in South Korea

**DOI:** 10.1017/dmp.2020.374

**Published:** 2020-10-12

**Authors:** Sukhyun Ryu, Youngsik Hwang, Hongbi Yoon, Byung Chul Chun

**Affiliations:** Department of Preventive Medicine, Konyang University College of Medicine, Daejeon, Republic of Korea; Department of Preventive Medicine, Korea University College of Medicine, Seoul, Republic of Korea

**Keywords:** compliance, coronavirus, COVID-19, Korea, quarantine

## Abstract

**Objectives:**

In South Korea, many individuals were self-quarantined for the coronavirus disease 2019 (COVID-19) after the quarantine criteria were extended to all overseas travelers. This study was conducted to identify the noncompliance rate of self-quarantine for COVID-19 cases and assess the impact of a 1-strike out policy and an increased amount of penalty for the violating self-quarantine in South Korea.

**Methods:**

The self-quarantine noncompliance rate for COVID-19 was examined using publicly available data. We collected the daily number of quarantine and quarantine violation cases from March 22 to June 10, 2020. A Poisson regression analysis was conducted to identify the impact of additional sanctions for the quarantine violation.

**Results:**

The median number of individuals quarantined per day was 36,561 (interquartile range, 34,408-41,961). The median number of daily self-quarantine violations was 6 (range, 0-13). The median rate of self-quarantine violations was 1.6 per 10,000 self-quarantined individuals (range, 0.0-8.0 per 10,000 self-quarantined individuals). The additional sanction has no significant impact on the number of violations among quarantine individuals (*P* = 0.99).

**Conclusions:**

The additional sanction for the violation of quarantined individuals did not reduce the self-quarantine violations. Further studies are warranted to strengthen the compliance of self-quarantine for future pandemics.

The severe acute respiratory syndrome coronavirus 2 (SARS-CoV-2) has affected South Koreans since January 20, 2020.^1^ To prevent the spread of SARS-CoV-2, the Korean government has implemented an unprecedented, large-scale home-quarantine for suspected or confirmed cases of coronavirus disease 2019 (COVID-19).^2^ In the early epidemics of COVID-19, financial aids to the those quarantined cases and compensation for the employer for the wage loss were provided under the Korean Infectious Disease Control and Prevention Act (Table 1).^3^ In South Korea, to prevent seeding new SARS-CoV-2 infection, any individual who has traveled from a country with a high COVID-19 infection risk is also obliged to self-quarantine.^4^ The quarantined individuals are asked to comply with self-quarantine and are monitored for 14 days. Since April 1, 2020, all overseas travelers have been included in the self-quarantine program, which has greatly increased the number of quarantined individuals in South Korea (Figure 1A). The self-quarantine individuals have been actively monitored by a mobile application or phone-call twice a day and the public health workers sometimes randomly visit the quarantined place in person (Table 1).^5^ On April 5, 2020, the Korean government implemented a “1-strike out policy” and increased the penalty from a maximum of 3 million to 10 million Korean Won (8273 USD) in fines to further discourage quarantine violation. A previous study demonstrated that self-quarantine compliance for the case of COVID-19 is crucial for preventing local transmission^6^; however, there is no literature measuring the exact noncompliance rate of self-quarantine for COVID-19 cases.

**TABLE 1:** Overview of the Korean strategy for encouraging self-quarantine for COVID-19 cases

Perspectives	Specific measures	Authorities
Support	• Korean public health authorities provide quarantined individuals, identified by contact tracing from the domestic transmission, with daily necessities and sanitary kits, worth around 60 USD, during their self-quarantine period.	• Local public health authorities • Local administrative government officials
	• The Korean government has provided financial aid totaling 374 USD per individual during the quarantine period.	• Ministry of the Interior and Safety • Local administrative government officials
	• Local governments provide individuals from overseas with transportation to a quarantine location when they arrive at the South Korean port of entry.	• Local administrative government officials
Monitoring	• A mobile application developed by the Korean government can be installed on quarantined individuals’ mobile phones. Quarantined individuals provide their location data and health status to nearby public health workers.	• Ministry of the Interior and Safety • Local public health authorities
	• Authorities have been conducting random on-site visits to self-quarantined individuals.	• Ministry of the Interior and Safety • Local public health authorities • Local police agencies
Sanctions	• On April 5, 2020, a 1-strike-out policy was implemented for all quarantined individuals. Korean public health authorities have enhanced noncompliance penalties. Those committing self-quarantine violations, such as an unauthorized breakaway, face up to on a year in prison or up to 10 million Korean won (8,273 USD) in fines under the Korean law to prevent and manage infectious diseases.	• Ministry of Justice • Ministry of the Interior and Safety
	• Foreign individuals who violate self-quarantine are subject to deportation.	• Ministry of Justice • Korea Immigration Services
	• Since April 27, 2020, Korean public health authorities have asked Koreans who violate the home quarantine to wear electronic bracelets.	• Ministry of the Interior and Safety • Local administrative government officials

**FIGURE 1:**
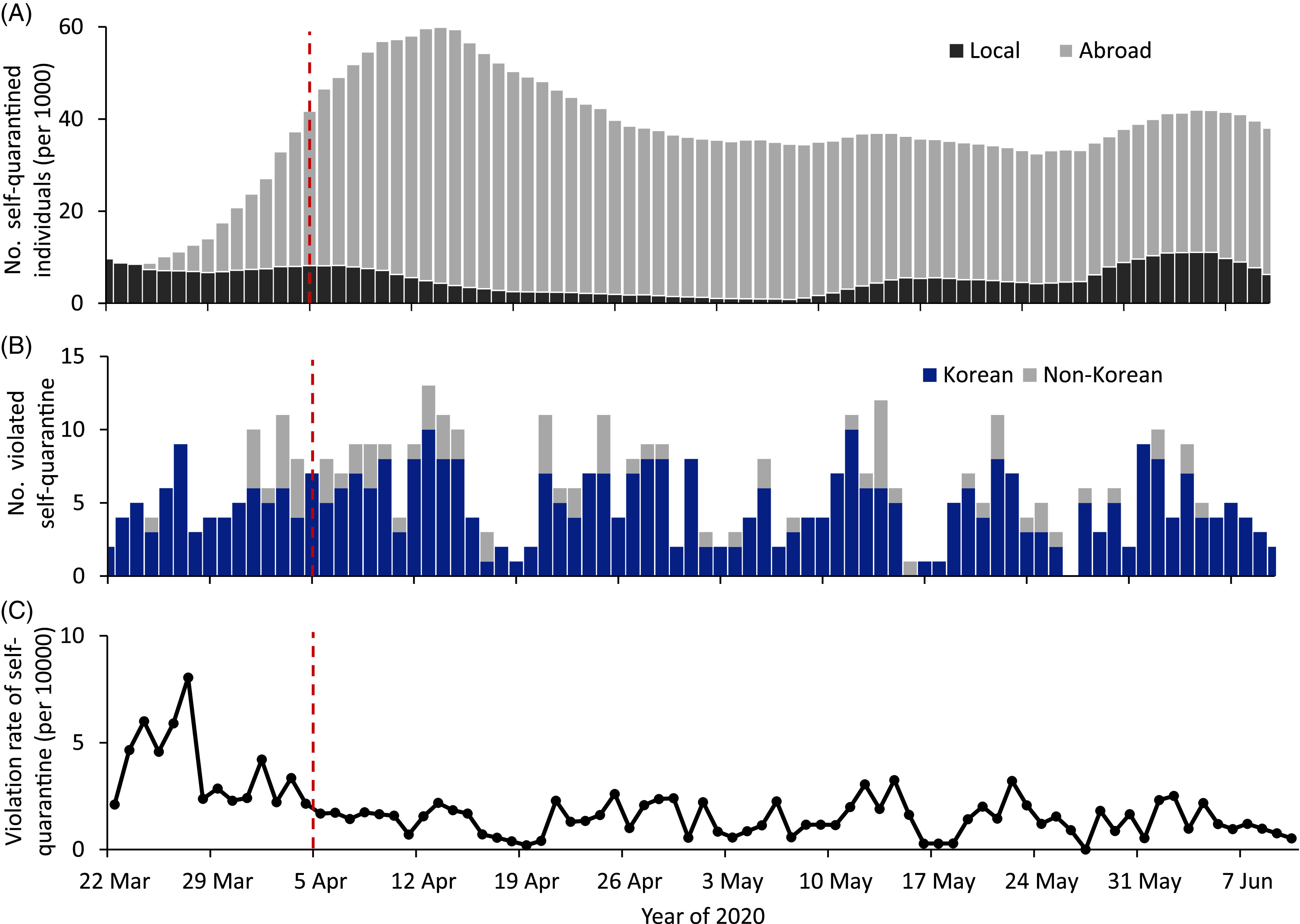
The Number of Individuals Quarantining and Individuals Who Have Violated Quarantine in South Korea Between March 22 and June 10, 2020. The vertical dashed line in red indicates the implementation of a 1-strike out policy and an increased penalty for the violation of individuals from self-quarantine. (A) The daily number of quarantined individuals as determined by contact tracing of locals and international travelers. (B) The daily number of violators. (C) The daily noncompliance rate of those in self-quarantine.

## METHODS

This study did not require the institutional review board approval or informed consent, because the data had already been published by the Korean Ministry of Interior and Safety.^7^ To obtain the noncompliance rate of self-quarantine for COVID-19 cases in South Korea, we collected the daily number of quarantine and quarantine violation cases from March 22 to June 10, 2020. To assess the impact of a 1-strike out policy and increased the penalty, the Poisson regression model incorporated with the additional sanctions were used to estimate the intervention effect with the confidence intervals on the daily rate (per 10,000 quarantined cases) and the daily number of quarantine violation. The established Poisson regression model is
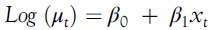



Where, μ_0_ represents the count of quarantine violation at day *t*, *β*_0_ and *β*_1_ denote estimate for base level of the quarantine violation and multiplicative effect on the quarantine violation, and *x_t_* represents the additional sanctions (*x_t_* = 0 from March 22 to April 5, 2020; *x_t_* = 1 from April 6 to June 10, 2020). All statistical analyses were performed in R version 3.0.2 (R Foundation for Statistical Computing, Vienna, Austria).

## RESULTS

The median number of individuals quarantined per day was 36,561 (interquartile range [IQR], 34,408-41,961; range, 8335-59,918). The median number of quarantined individuals, including those in contact with suspected and confirmed cases and those who have traveled overseas, was 5127 (IQR, 2451-7568; range, 846-11,053) and 32,041 (IQR, 28,923-37,819; range, 0-55,590), respectively (Figure 1A). The median number of daily self-quarantine violations was 6, with a range of 0-13 (Figure 1B). The median rate of self-quarantine violations was 1.6 per 10,000 quarantined individuals (range, 0.0-8.0 per 10,000) (Figure 1C). We identified a 1-strike out sanction and increased the amount of penalty had not a significant change on the daily violation rate (*P* = 0.99) (Table 2). Furthermore, it had not any significant impact on the number of violations among Koreans (*P* = 0.91) and foreigners (*P* = 0.81).

**TABLE 2:** Results of a Poisson regression analysis by implementation of a 1-strike out policy and increased the amount of penalty

Parameter	Estimates	95% Confidence Interval	p-value
Rate of quarantine violation^a^	0.001	−0.23 - 0.25	0.99
Number of quarantine violation of Koreans	0.02	−0.24 - 0.29	0.91
Number of quarantine violation of foreigners	−0.07	−0.59 - 0.53	0.81

a rate: per 10,000 quarantined individuals

## DISCUSSION

To limit the local transmission of SARS-CoV-2, public compliance with public health measures, such as self-quarantine for suspected or confirmed cases of COVID-19 is essential.^6,8^ A previous study demonstrated that a major obstacle to self-quarantine compliance was concern over the loss of income.^9^ To overcome this obstacle, the Korean government developed 3 specific plans early in the epidemic to reimburse, monitor, and sanction the individuals in self-quarantine for COVID-19 (Table 1).

The Korean government’s efforts to control the local transmission of SARS-CoV-2 has been positively evaluated among global public health experts; however, the implemented policies, particularly the forced control of people, is necessary to discuss. Our findings indicate that additional self-quarantine regulations did not significantly affect the violation rate of quarantine. This signifies the importance of a proper level of policy execution to control the number of violations and helps to decrease unnecessary costs and avoid time-consuming decision-making processes. Our findings also provide a proxy of the self-quarantine noncompliance rate under governmental regulation. However, this study is limited by a lack of demographic data and details on the reasons for violations, which make it challenging to identify the risk factors associated with breaches of self-quarantine rules. Furthermore, additional time-series data before our study period are required to improve the model fit and accuracy.

As of September 7, 2020, the two COVID-19 epidemic waves identified,^10^ and the violations of self-quarantine are still being reported in South Korea. Further studies including the effective process for delivering comprehensive information about the self-quarantine to the public and appropriate level of policy for discouraging self-quarantine violations are warranted.

## CONCLUSIONS

Our results identified the additional sanctions, including a 1-strike out policy and increased the amount of penalty, had no significant impact on reducing the violation rate of self-quarantine in South Korea. Additional studies are warranted to strengthen quarantine compliance for future epidemics.

## Data Availability

The study data are available from the corresponding author upon reasonable request.
